# Chronic Subdural Hematoma in a Middle-Aged Amateur Scuba Diver: A Case Report

**DOI:** 10.7759/cureus.56049

**Published:** 2024-03-12

**Authors:** Sayaka Ito, Yoshinori Maki, Kazushi Higuchi

**Affiliations:** 1 Neurosurgery, Kohka Public Hospital, Koka, JPN; 2 Neurosurgery, Hikone Chuo Hospital, Hikone, JPN; 3 Rehabilitation, Hikari Hospital, Otsu, JPN; 4 Neurosurgery, Japanese Red Cross Nagahama Hospital, Nagahama, JPN

**Keywords:** chronic subdural hematoma (csdh), intracranial hemorrhage, scuba diving, decompression sickness, barotrauma

## Abstract

Scuba diving has become a common and popular activity, and adverse events can occur following this activity. Among those events, intracranial hemorrhage is very rare, and only intracerebral hemorrhage and subarachnoid hemorrhage are reported. However, the occurrence of chronic subdural hematoma (CSDH), possibly as an adverse event following scuba diving, has not been described. A 49-year-old man with no significant medical history visited our hospital complaining of memory disturbance and aphasia. He had experienced a minor head trauma five months before and had gone scuba diving six times between the traumatic episode and the visit to our hospital. A brain computed tomography scan revealed a left CSDH. The patient underwent burr-hole surgery to remove the CSDH, and his symptoms resolved. We report the first case of CSDH possibly related to scuba diving. No recurrence of CSDH was observed at 28 months postoperatively.

## Introduction

Scuba diving has become a worldwide activity in recent decades [1]. Due to the physical stress related to the drastic change in ambient pressure during or after this activity, adverse events can occur [2,3]. In general, severe injury and death are uncommon in scuba diving accidents [1]. However, some pathophysiological challenges may lead to a variety of unusual diseases, where rapid diagnosis and treatment are critical [3]. The behavior of gases under changing pressure conditions, known as barotrauma and decompression sickness, is a mechanism involved in dive-related complications [3]. The nervous system is frequently affected by complications and fatalities related to scuba diving [1]. However, intracranial hemorrhagic events, including intracerebral hemorrhage and subarachnoid hemorrhage after scuba diving, are rare [4-6]. Besides, to the best of our knowledge, there has not been any description concerning chronic subdural hematoma (CSDH) as an adverse event after scuba diving.

Here, we report a rare case of CSDH as an adverse event following scuba diving.

## Case presentation

A 49-year-old man with no significant medical history visited our hospital with complaints of gradual memory disturbance and aphasia for several days. The patient did not take any medication. He had no family history of hematologic diseases. He had experienced a minor traffic accident and had hit his head against the headrest without any other physical trauma five months before but did not require any medical treatment. After that episode, he went scuba diving three times in total: two, three, and four months before the visit. He dived for 70 minutes, reaching 30 m below the water’s surface twice every time. The patient experienced no loss of consciousness or other physical or neurological problems during and after the activity. During the episode of head trauma and the visit to our hospital, he had not had any complaints or experienced any physical trauma on any occasion, including scuba diving. On arrival at our hospital, he was alert but had right upper limb paralysis. Laboratory tests, including blood cell counts and biochemical and coagulation tests of blood, were normal (Table 1).

**Table 1:** Initial blood cell counts and biochemical and coagulation results for the case AST, aspartate aminotransferase; ALT, alanine aminotransferase; γ-GTP, γ-glutamyl transpeptidase; LDH, lactate dehydrogenase; BUN, blood urea nitrogen; CRP, C-reactive protein; PT, prothrombin time; aPTT, activated partial thromboplastin time

Test (unit)	Patient’s data	Reference range
Total protein (g/dL)	6.6	6.7-8.3
Albumin (g/dL)	4.00	3.8-5.3
Total bilirubin (ｍg/dL)	0.6	0.2-1.0
AST (IU/L)	13	9-36
ALT (IU/L)	7	5-42
γ-GTP (IU/L)	11	13-64
LDH (IU/L)	142	124-222
Amylase (IU/L)	89	44-132
BUN (ｍg/dL)	14.4	8.1-21.9
Creatinine (ｍg/dL)	0.90	0.66-1.08
Uric acid (ｍg/dL)	6.2	2.4-7.0
Sodium (mEq/L)	141.8	137-148
Potassium (mEq/L)	3.72	3.50-5.10
Chloride (mEq/L)	106.3	100-110
Glucose (ｍg/dL)	93	none
CRP (ｍg/dL)	0.06	0.0-0.5
White blood cells (×10^3^/µL)	6.9	3.5-9.5
Red blood cells (×10^6^/µL)	4.68	4.10-5.30
Hemoglobin (g/dL)	13.9	13.6-17.0
Hematocrit (%)	41.3	40-52
Platelets (×10^3^/µL)	227	150-350
PT (sec)	11.3	10-13
aPTT (sec)	27.5	25-40
Fibrinogen (ｍg/dL)	336	151-398

**Figure 1:**
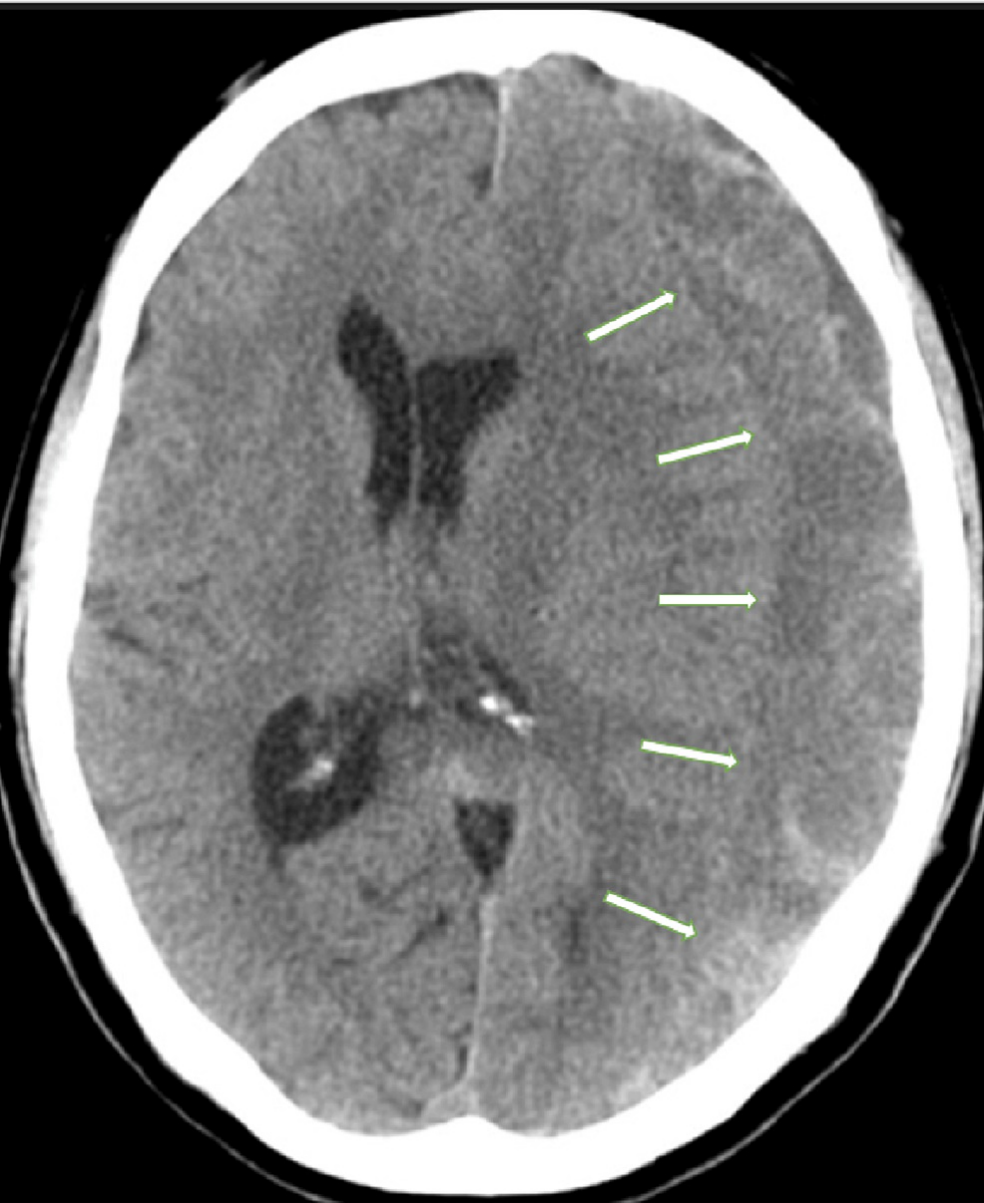
Brain computed tomographic findings A left chronic subdural hematoma is identified. The hematoma shows a hetero-density signal (arrows).

A brain computed tomography (CT) scan to rule out an intracranial lesion revealed a left CSDH (Figure 1).

The CSDH showed a hetero-intensity signal. The neurological symptoms in the patient seemed to have resulted from that lesion. The patient underwent burr-hole surgery to remove the CSDH. His symptoms resolved immediately after the surgery, and he was discharged the following day with no neurological deficits. No recurrence of CSDH was observed 28 months after surgery. The patient recovered fully and did not have any symptoms.

## Discussion

We report a rare case of CSDH following scuba diving. After a minor head trauma and repeated scuba diving activity, a CSDH became symptomatic, and surgical removal was warranted. This is the first report concerning the formation of CSDH as an adverse effect related to scuba diving.

Typical adverse events during or after scuba diving are known as barotrauma and decompression sickness [2]. Barotrauma refers to pressure-related tissue damage in divers caused by variations in air pressure and is explained by Boyle’s law, which states that the volume of gas varies inversely with the pressure applied [3]. Gas bubble-induced endothelial injury after scuba diving has been observed in animal studies [7] and is thought to be a major mechanism of decompression sickness. Gas bubbles (usually nitrogen or helium) are known to spontaneously form in water containing dissolved gas [8]. This phenomenon might occur in scuba diving, where an ambient pressure gradient exists, especially with rapid decompression. Scuba divers can experience endothelial damage associated with scuba diving. We hypothesize that our patient might have experienced diving-associated endothelial damage to the bridging veins and/or feeders of subdural membranes, which might have progressed to the CSDH. In addition to decompression sickness, increasing intracranial pressure induced by high ambient pressure conditions during scuba diving [9] may result in the formation of intracranial hemorrhage.

There are only limited reports of intracranial hemorrhage associated with scuba diving. Reichardt et al. reported a case of a 47-year-old man who experienced severe headache and loss of consciousness after an emergency ascent from 26 m depth following a 42-minute scuba dive [6]. His condition resulted from an aneurysmal subarachnoid hemorrhage. The authors hypnotized that subarachnoid hemorrhage could have been caused by barotrauma or elevated intracranial pressure [6]. Kohshi et al. presented two cases of intracranial hemorrhage during and after diving [4]. The first case was a 45-year-old woman who experienced a sudden severe headache 30 minutes after appropriate ascension from a 20 m depth following a 50-minute scuba dive. The patient was diagnosed with subarachnoid hemorrhage with a brain CT scan. The second case was of a 49-year-old fisherman who developed right hemiparesis and speech difficulty during scuba diving. A brain CT scan revealed a left putaminal hemorrhage. In both cases, there was no detailed description of the link between diving and hemorrhage [4]. Piper et al. reported a case of a 60-year-old man who presented with mental confusion, headache, and vomiting after scuba diving [5]. A brain CT scan revealed a subarachnoid hemorrhage. The authors proposed that the etiology of subarachnoid hemorrhage after diving might have been ischemic insults in combination with venous congestion caused by intravascular nitrogen bubbles after decompression sickness [5]. However, to the best of our knowledge, there have been no cases of CSDH related to scuba diving similar to our case (Table 2).

**Table 2:** Published case reports of scuba diving-related intracranial hemorrhage HA, headache; LOC, loss of consciousness; SAH, subarachnoid hemorrhage; CSDH, chronic subdural hematoma; ICP, intracranial pressure; N/A, not assessed

Author, year [reference no.]	Age/sex	Symptoms	Types of hemorrhage	Probable cause
Reichardt et al., 2003 [6]	47/M	HA LOC	SAH	Barotrauma Elevated ICP
Kohshi et al., 2017 [4]	45/F	HA	SAH	N/A
	49/M	Hemiparesis aphasia	Putaminal hemorrhage	N/A
Piper et al., 2020 [5]	60/M	Mental confusion HA vomiting	SAH	Ischemic insults and venous congestion caused by intravascular nitrogen bubbles
Our case	49/M	Memory disturbance aphasia	CSDH	Previous head trauma, endothelial damage to the bridging veins and/or feeders of subdural membranes, and elevated ICP

In our case, the preceding head trauma could have been a trigger for the formation of CSDH. Following this episode, repeated scuba diving could have contributed to the growth of CSDH. This hypothesis seems to correspond to the radiographic finding that the CSDH showed a hetero-signal intensity; the CSDH grew gradually with the repeated intermittent intracranial pressure change. As this is the first report on CSDH that is possibly associated with scuba diving, our experience should be shared with clinicians.

## Conclusions

We describe the first case of CSDH after scuba diving. In our case, a preceding minor head trauma and several times of scuba diving activity might have resulted in the formation of CSDH. Hypothesized induction of elevated intracranial pressure and/or endothelial damage related to scuba diving could have triggered the formation and/or progression of CSDH. Although this is just a report of one case, our case should be shared among clinicians as a rare adverse event after scuba diving.

## References

[1] Newton HB (2001). Neurologic complications of scuba diving. Am Fam Physician.

[2] Gorman DF (1989). Decompression sickness and arterial gas embolism in sports scuba divers. Sports Med.

[3] Melamed Y, Shupak A, Bitterman H (1992). Medical problems associated with underwater diving. N Engl J Med.

[4] Kohshi K, Morimatsu Y, Tamaki H, Murata Y, Kohshi K, Ishitake T, Denoble PJ (2017). Cerebrospinal vascular diseases misdiagnosed as decompression illness: the importance of considering other neurological diagnoses. Undersea Hyperb Med.

[5] Piper K, Screven R, Agazzi S, Guerrero WR, Dombrowski K (2020). Nonaneurysmal subarachnoid hemorrhage in scuba diving. World Neurosurg.

[6] Reichardt KA, Nabavi A, Barth H, Mehdorn HM, Blömer U (2003). Barotrauma as a possible cause of aneurysmal subarachnoid hemorrhage. Case report. J Neurosurg.

[7] Arieli R, Arieli U, Marmur A (2015). Bubble size on detachment from the luminal aspect of ovine large blood vessels after decompression: the effect of mechanical disturbance. Respir Physiol Neurobiol.

[8] Arieli R (2017). Nanobubbles form at active hydrophobic spots on the luminal aspect of blood vessels: consequences for decompression illness in diving and possible implications for autoimmune disease-an overview. Front Physiol.

[9] Mehrpour M, Shams-Hosseini NS, Rezaali S (2014). Effect of scuba-diving on optic nerve and sheath diameters. Med J Islam Repub Iran.

